# Vitrification: pursuing technologies to improve safety and efficacy

**DOI:** 10.1016/j.xfre.2024.02.006

**Published:** 2024-02-15

**Authors:** Richard T. Scott

**Affiliations:** a Foundation for Embryonic Competence, Basking Ridge, New Jersey; b Department of Biomedical Sciences, University of South Carolina-Greenville School of Medicine, Greenville, South Carolina; c Division of Reproductive Endocrinology and Infertility, Department of Obstetrics and Gynecology, Yale University, New Haven, Connecticut

This issue of *Fertility and Sterility Reports* includes a contribution by Hristova et al. (1) regarding a potentially important alternate method for blastocyst vitrification. The technique achieves the ultrarapid cooling requisite for vitrification by immersing the embryo into precooled aluminium oxide (Al_2_O_3_) powder instead of directly into liquid nitrogen (N_2_). This pilot clinical study found that enhanced outcomes were attained using the new technique, providing an exciting impetus for more comprehensive and robust clinical studies in the near future.

Vitrification is an important clinical technique capable of producing excellent results and has been widely adopted by clinical assisted reproductive technology laboratories. The blastocyst survival rates of ≥95% are routinely achieved within several clinical programs. Although contemporary results are dramatically better than those attained with conventional “slow” cryopreservation protocols, they remain imperfect. There are also issues beyond simple survival. The need to maximize cooling gradients to attain such high survival rates has led some embryologists to use open systems during the vitrification process. This creates a putative risk of infection of an embryo because viruses have been isolated in liquid N_2_. The technique also requires great technical expertise and may be sensitive to even minor drifts in technique.

Understanding the potential impact of using Al_2_O_3_ powder as the cooling agent in blastocyst vitrification requires some understanding of the basic aspects of cellular vitrification. When mammalian cells are cooled sufficiently rapidly to a critical threshold, they form a hyperviscous liquid (a vitreous) instead of crystalline solids. Because significant numbers of ice crystals are lacking, there is no increase in the volume, and there is no bursting of cells or the organelles within them.

However, the glass transition temperature (vitrified materials are in a glass-like state) for mammalian cells is in the −130°C range (the exact temperature depends on cryoprotectants, pressure, and cooling rate). This is considerably colder than the temperature where ice crystals begin to form within cells, which is approximately −8°C. This creates a seeming paradox where cooling to the temperatures required for vitrification requires the embryo first be at substantial risk of harmful ice crystal formation. Cryoprotectants are used to help attenuate ice formation within the cells and, when combined with ultrarapid cooling rates, will cool the embryo to the glass transition temperature before impactful ice crystal formation. Practical limits on attaining vitrification are controlled by the maximum rate of cooling (faster is better) and the concentration of cryoprotectants before they demonstrate toxicity.

There is more to ultrarapid cooling than just the magnitude of the temperature gradient and thermal conductivity. The *Leidenfrost effect*, which the investigators importantly refer to in their publication, is the key (2). When a cool liquid (the liquid N_2_) is exposed to a much warmer substance (the blastocyst and cryodevice), thermal energy is rapidly transferred from the warm substance to the much cooler liquid N_2_. Within that microenvironment, the temperature of the liquid N_2_ increases rapidly and literally boils, forming N_2_ gas. The N_2_ gas coalesces at the junction of the warmer material (the embryo and cryodevice) and the liquid N_2_, forming a thin gaseous film (Fig. 1). Indeed, this is known as thin film boiling. The impact of this thin film of gas depends on several factors but may reduce thermal transfer rates (i.e., cooling) by an order of magnitude or more. Minimizing this effect requires a stringent and precise technique during the vitrification process, and the much slower cooling rates may require the use of higher and more toxic levels of cryoprotectants.

**Figure 1:**
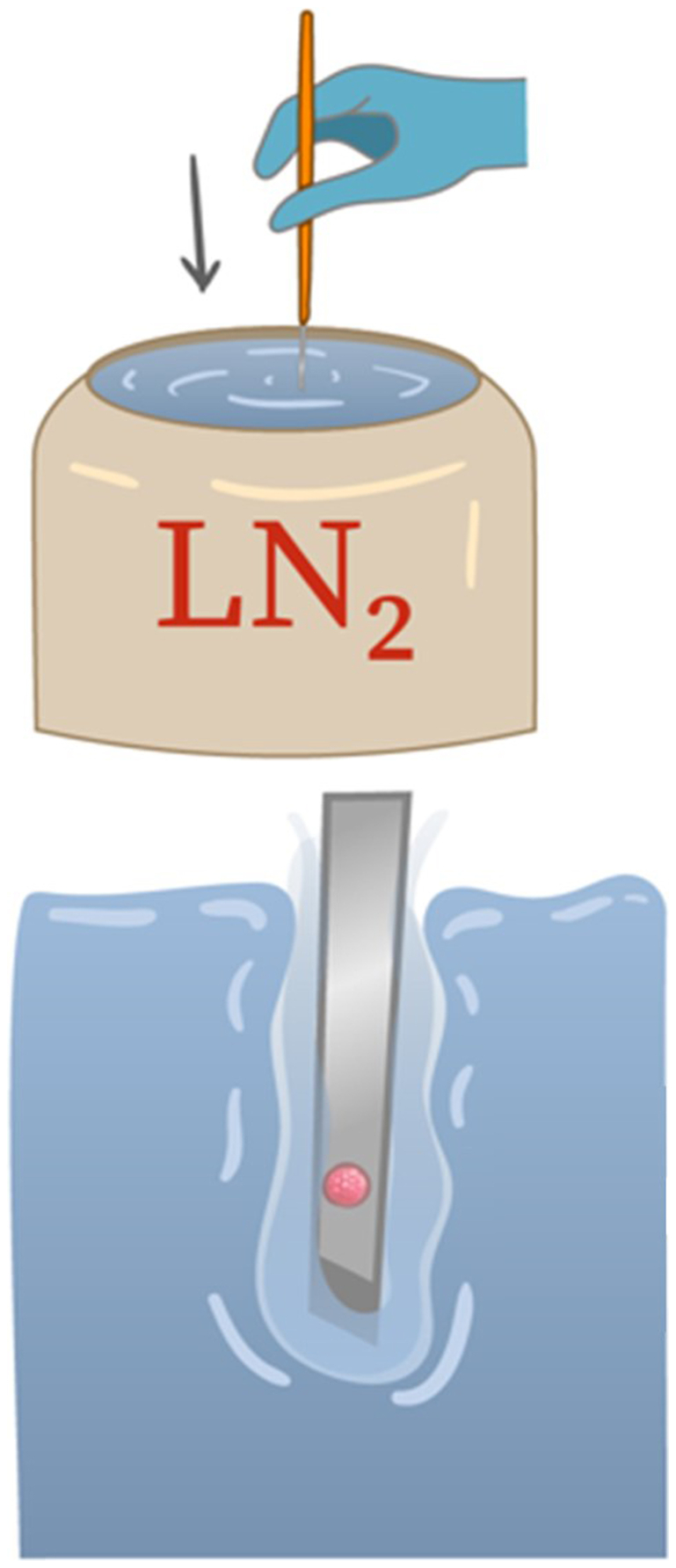
When a warm blastocyst and cryosystem were plunged into liquid nitrogen (N_2_), the adjacent liquid N_2_ boiled, leading to the formation of a thin film of N_2_ gas. This gas functionally insulated the embryo from the liquid N_2_ and, thus, may markedly diminish the rate of cooling. The reduced cooling rate may allow ice crystal formation, and the embryo may be damaged. Alternatively, very high and potentially toxic levels of cryoprotectants were used to reduce that risk. (Artwork by Saxon Scott)

So how may the approach advocated by Hristova et al. (1) improve vitrification outcomes? The expectation of improved performance is not intuitive. To begin with, the technique uses the same concentrations and schedules of cryoprotectant exposure. Liquid N_2_ is used to precool the metal device that contains the Al_2_O_3_ powder. Thus, both are −196°C when the vitrification process begins—supplying identical initial cooling gradients. However, the thermal energy transfer rate is faster to Al_2_O_3_ than to liquid N_2_ (30 vs. 25 Wm^−1^ K^−1^), which should result in a faster cooling rate. Although the difference is modest, it is possible that survival be impacted in some embryos.

Most important is that the technique described by Hristova et al. (1) provides an elegant solution to overcome the Leidenfrost effect. The powder is a solid, and thus, there is nothing to boil because it absorbs energy from the embryo. No gas forms; therefore, there is no thin film barrier. Cooling should occur more predictably and more rapidly and be less dependent on the operator technique.

Will this new technique meaningfully improve survival after vitrification? The probable answer is no. Survivals in several programs already exceed 98%; thus, opportunity for enhancing outcomes is minimal. A massive sample size of 4,368 patients would be required for a randomized controlled trial, which would demonstrate a 1% improvement in outcomes. Such a study is impractical. However, other important benefits may accrue. The faster and more reliable cooling may allow the effective use of closed cryosystems. These systems have more mass to cool and invariantly have some sort of barrier between the cooling substrate and embryo, which may limit the rate of cooling for the embryo. The lack of the Leidenfrost effect and the faster thermal transfer rate may overcome this disadvantage. This would add an element of safety because the embryo would never be exposed to liquid N_2_ and the putative infectious risks that have been hypothesized. The system may tolerate larger variances in technique during the vitrification process. This would simplify procedures in the laboratory and may even allow for more robust automated systems in the future.

Several additional studies will be required to fully understand this approach to blastocyst vitrification and its role in clinical practice. Although improvement in survival would be wonderful, increases in safety and efficiency could show the technique to be useful either way. This pilot study will capture the interest of several embryologists and clinical scientists. Hopefully, high-quality and adequately powered studies will follow soon.

## CRediT Authorship Contribution Statement

**Richard T. Scott:** Conceptualization, Writing – original draft, Writing – review & editing.

## Declaration of Interests

R.T.S. has nothing to disclose.
